# NiCoP/g-C_3_N_4_ Nanocomposites-Based Electrochemical Immunosensor for Sensitive Detection of Procalcitonin

**DOI:** 10.3390/s23094348

**Published:** 2023-04-28

**Authors:** Furong Chen, Layue Bao, Ying Zhang, Ruili Wang, Jinghai Liu, Wenfeng Hai, Yushuang Liu

**Affiliations:** 1 Inner Mongolia Key Laboratory of Carbon Nanomaterials, Nano Innovation Institute (NII), College of Chemistry and Materials Science, Inner Mongolia Minzu University, Tongliao 028000, China; 2 College of Bioengineering, Beijing Polytechnic, Beijing 100176, China

**Keywords:** procalcitonin, NiCoP/g-C_3_N_4_ nanocomposites, electrochemical immunosensor, human serum blood

## Abstract

Herein, an ultra-sensitive and facile electrochemical biosensor for procalcitonin (PCT) detection was developed based on NiCoP/g-C_3_N_4_ nanocomposites. Firstly, NiCoP/g-C_3_N_4_ nanocomposites were synthesized using hydrothermal methods and then functionalized on the electrode surface by π-π stacking. Afterward, the monoclonal antibody that can specifically capture the PCT was successfully linked onto the surface of the nanocomposites with a 1-(3-Dimethylaminopropyl)-3-ethylcarbodiimide hydrochloride (EDC) and N-Hydroxysuccinimide (NHS) condensation reaction. Finally, the modified sensor was employed for the electrochemical analysis of PCT using differential Pulse Voltammetry(DPV). Notably, the larger surface area of g-C_3_N_4_ and the higher electron transfer capacity of NiCoP/g-C_3_N_4_ endow this sensor with a wider detection range (1 ag/mL to 10 ng/mL) and an ultra-low limit of detection (0.6 ag/mL, S/N = 3). In addition, this strategy was also successfully applied to the detection of PCT in the diluted human serum sample, demonstrating that the developed immunosensors have the potential for application in clinical testing.

## 1. Introduction

Procalcitonin (PCT), an environmental-friendly biomarker of a variety of diseases induced by bacterial infections, has already played a significant role in clinical medicine practice [1,2,3,4,5]. Currently, various detection methods have been developed for PCT quantitative detection, such as enzyme-linked immunosorbent assays (ELISA) [6,7], protein microarrays [8,9,10], electrochemiluminescent immunosensors [11,12,13], surface plasmon resonance biosensors [14,15,16] and electrochemical biosensors [17,18,19]. Compared with other methods, the electrochemical biosensor is an effective analytical approach, and it has attracted great interest owing to its advantages of high sensitivity and selectivity, rapid response, low cost and easy miniaturization [20,21,22]. However, choosing the right electrode material remains a challenge for achieving a high-performance electrochemical sensor.

In recent years, nanocomposites based on graphene and its derivatives have drawn considerable attention in the field of sensing. Graphite-like carbon nitride (g-C_3_N_4_), a two-dimensional planar conjugation material, because of its larger surface area, unique electronic band structure, good biocompatibility, excellent electronic and physicochemical properties, has been taken as a promising alternative for SERS sensing and bioimaging, optoelectronics, direct solar water splitting and visible light photocatalytic pollutant degradation [23,24,25,26,27]. Despite these excellent properties, the application of g-C_3_N_4_ in the electrochemical sensing area is still restricted because of the large contact resistance and poor conductivity. To overcome this disadvantage, some alterations, such as doping or coupling with other nanomaterials, were adopted to improve the conductivity, making it suitable for electrochemical sensor applications.

Transition metal phosphides, such as CoP, Cu_3_P and Ni_2_P, because of their quasi-metallic properties, good conductivity, stability and high electrocatalytic activity, have been widely used in photocatalysis, supercapacitors and other research fields. However, as an electrochemical material, due to the synergy between metals, bimetallic phosphide nanomaterials showed better electrochemical properties than the corresponding monometallic [28]. Additionally, features such as high electrical conductivity, the low electronegativity of phosphorus compared to oxygen, and less negative charge of phosphorus make them particularly suitable for biosensor design. The ternary metal phosphide NiCoP has received significant attention in the field of electrocatalysis for its excellent electrical conductivity and electronic structure. In addition, factors such as a low overpotential and small charge transfer impedance make NiCoP exhibit better electrochemical performance than single [29,30,31,32]. However, to the best of our knowledge, there has been no research on using NiCoP as a sensing interface to improve sensing performance.

**Scheme 1:**
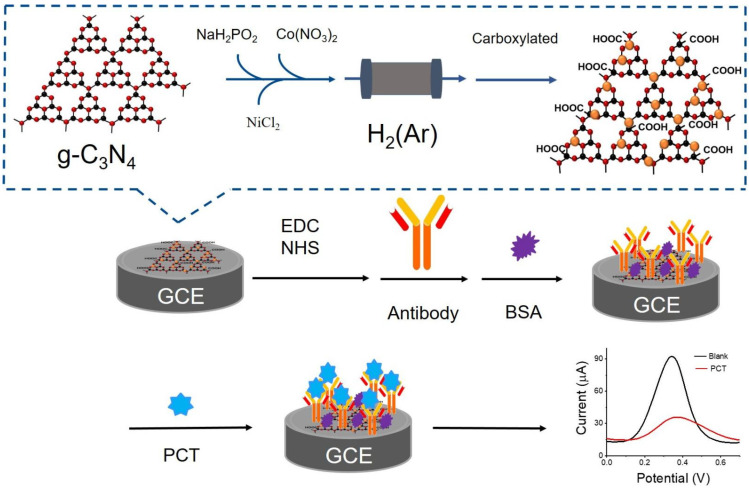
Schematic illustration of the NiCoP/g-C_3_N_4_ nanocomposites-based electrochemical immunosensor for detecting PCT. The preparation process of the NiCoP/g-C_3_N_4_ and COOH-NiCoP/g-C_3_N_4_ was shown in dashed line box.

In this paper, a new electrochemical immunosensor based on NiCoP/g-C_3_N_4_ nanocomposite modification was for the first time constructed and used for PCT detection (Scheme 1). The formation of the NiCoP/g-C_3_N_4_ nanocomposites was examined by X-ray photoelectron spectroscopy (XPS), X-ray diffractometry (XRD), scanning electron microscope (SEM), and transmission electron microscopy (TEM), and the functionalization was confirmed using Fourier transform infrared spectroscopy (FTIR). Additionally, the electrochemical properties of this sensing platform were investigated in monitoring the PCT with different concentrations and with different backgrounds. The recovery of this sensing platform was investigated by monitoring different pulse voltammetry (DPV) of PCT in the presence of human serum blood.

## 2. Experiment Section

### 2.1. Materials

Nickel (II) Chloride Hexahydrate Puratrem (NiCl_2_·6H_2_O), Cobaltous Nitrate Hexahydrate (CoH_12_N_2_O_12_), Sodium hypophosphite monohydrate (NaH_2_PO_2_·H_2_O) and urea were purchased from Tansoole (Shanghai, China). The PCT, monoclonal antibody, Interleukin-6 (IL-6), and C-reactive protein (CRP) were purchased from Cusabio Biotech CO. (Wuhan, China). Tris-HCl buffer was purchased from Solarbio life sciences (Beijing Solarbio Science & Technology Co., Beijing, China).

### 2.2. Instrumentations

The structure of the nanocomposites samples was analyzed using transmission electron microscopy (TEM) (JEOL, 200 KV, MA, USA), scanning electron microscopy (SEM) (S-4800, Hitachi, Tokyo, Japan), X-ray diffraction (XRD) (Smatlab, Tokyo, Japan) and Fourier transform infrared spectroscopy (FT-IR) (Nicolet, 6700, MA, USA). The elemental compositions were examined using X-ray photoelectron spectroscopy (XPS) (ESCALAB Xi+, Thermo Fisher Scientific, MA, USA). Electrochemical measurements were performed on multi-channel electrochemical work station (VMP3, Bio-Logic Science Instruments, Seyssinet-Pariset, France) with EC-Lab software version 11.10 (bio-logic, Seyssinet-Pariset, France).

### 2.3. Synthesis of g-C_3_N_4_ and NiCoP/g-C_3_N_4_ Nanocomposite

The preparation method of g-C_3_N_4_ followed previously published protocols [33]. Briefly, urea was put in a crucible with a cover under ambient pressure in air. When heated to 550 °C at a ramp rate of 5 °C/min and maintained in an Argon environment for another 3 h, the yellow powder was obtained.

The synthesis of NiCoP/g-C_3_N_4_ composite also followed previously published protocols [34]. Briefly, a quantity of NiCl_2_·6H_2_O (50 mg), CoH_12_N_2_O_12_ (50 mg) and Sodium NaH_2_PO_2_·H_2_O (50 mg) was dissolved in 10 mL Milli-Q water, respectively. The amount of 300 mg g-C_3_N_4_ was added to each solution and stirred 6 h after sonication for 1 h and dried by vacuum drying. The remaining power was ground and heated at 350 °C in an Argon environment for 2 h. A white powder NiCoP/g-C_3_N_4_ nanocomposite was obtained.

### 2.4. Carboxylation of NiCoP/g-C_3_N_4_ Nanocomposites (COOH-NiCoP/g-C_3_N_4_)

The 1 g NiCoP/g-C_3_N_4_ nanocomposite was ground and dissolved in 100 mL of HNO_3_. The mixture was heated in an oil bath at 125 °C for one day. After cooling down to room temperature, the solid substance was collected and vacuumed at 35 °C overnight.

### 2.5. Preparation of PCT Biosensor

The bare glass carbon electrode (GCE) was polished with an alumina slurry and washed with water, then 20 μL COOH-NiCoP/g-C_3_N_4_ suspension (1 mg COOH-NiCoP/g-C_3_N_4_ dispersed into 1 mL ethanol under sonication for 30 min) was dropped on its surface and incubated for 3 h. After that, 10μL EDC and NHS mixed solution, and 10 μL and 1 μM monoclonal antibody, respectively, were dropped on the electrode surface and incubated at room temperature. Finally, BSA (2%, *W*/*V*) was used to cover the non-specified binding sites of the electrode.

### 2.6. General Characterization

The electrochemical measurement was performed in 5 mM K_3_Fe(CN)_6_/K_4_Fe(CN)_6_ + 1× Tris-HCl buffer. Three electrodes consisting of a NiCoP/g-C_3_N_4_ nanocomposite modified working electrode, platinum wire counter electrode and Ag/AgCl reference electrode were used. FT-IR was collected with KBr pressed as pellets on a Nicolet 6700-IR spectrophotometer in the range 400–4000 cm^−1^. X-ray diffraction (XRD) patterns were collected on a SmartLab instrument equipped with graphite-monochromatized Cu Ka radiation (λ = 0.1541 nm; scan speed of 6 min^−1^; 2θ = 10–80°) at room temperature, with XPS using Al Kα radiation as the X-ray source (1486.7 eV) with the pass energy of 30 eV.

### 2.7. PCT Sensing in Tris-HCl 

PCT in a range from 1 ag/mL to 10 ng/mL in 1× Tris-HCl (pH 7.0) was measured by DPV in the 5 mM K_3_Fe(CN)_6_/K_4_Fe(CN)_6_. The glassy carbon electrode with NiCoP/g-C_3_N_4_ modified was acting as a working electrode, the counter electrode was platinum wire, and Ag/AgCl was the reference electrode. The mean and standard deviation were obtained from three replicated tests.

### 2.8. PCT Detection in Serum Sample

With the approval of the school ethics committee, the human blood samples were obtained from the Affiliated Hospital of Inner Mongolia Minzu University. Serum samples were centrifuged at 5000 rpm for 20 min and filtered with a 0.22 μm membrane prior to use. For the recovery test, the collected serum was diluted 100 times with Tris-HCl buffer, and then PCT was added to obtain the final concentration. The recoveries were determined from samples containing three concentrations of PCT in human serum.

## 3. Results and Discussion

### 3.1. The Morphology and Structural Information

**Figure 1:**
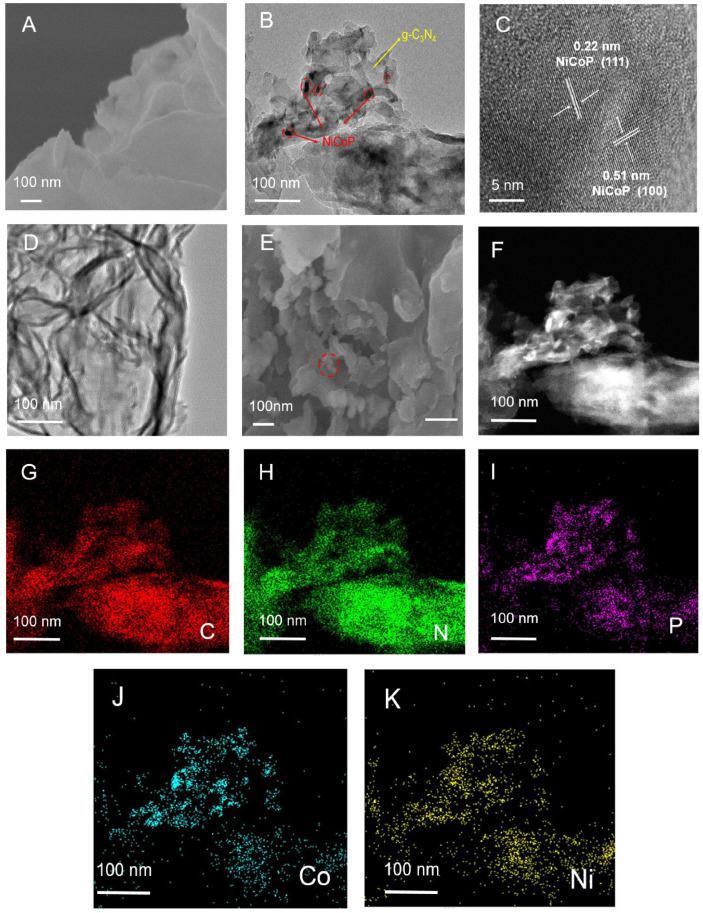
(**A**) SEM image of g-C_3_N_4_, (**B**) TEM image of NiCoP/g-C_3_N_4_, (**C**) Characteristic HRTEM image for a typical nanoparticle, (**D**) TEM image of g-C_3_N_4_. (**E**) SEM image of NiCoP/g-C_3_N_4_. (**F**) High-resolution STEM image, and elemental mappings of C (**G**), N (**H**), P (**I**), Co (**J**), Ni (**K**).

The morphologies of g-C_3_N_4_ and NiCoP/g-C_3_N_4_ nanocomposites were investigated by SEM and TEM. For the g-C_3_N_4_, they appeared layered, rippled and resembled waves of crumpled silk veil, illustrating the nanosheets structure (Figure 1A,D). Our previous research identified that this special nanosheet structure could provide more active binding sites [35]. For the NiCoP/g-C_3_N_4_ nanocomposites, there were several small nanoparticles displayed on the g-C_3_N_4_ surface, implying that NiCoP was successfully loaded onto the g-C_3_N_4_ surface (Figure 1B,E). The HRTEM image shows distinct lattice fringes with a plane spacing of 0.22 nm and 0.51nm, corresponding to the (111) and (100) planes of NiCoP (Figure 1C) [36,37,38]. In addition, the energy-dispersive X-ray spectroscopy (EDX) mapping image obtained in STEM mode demonstrated the C and N elements distributed in the substrate, and the Ni, Co and P elements throughout the structure (Figure 1F–K), further confirming the presence of NiCoP nanoparticles.

**Figure 2:**
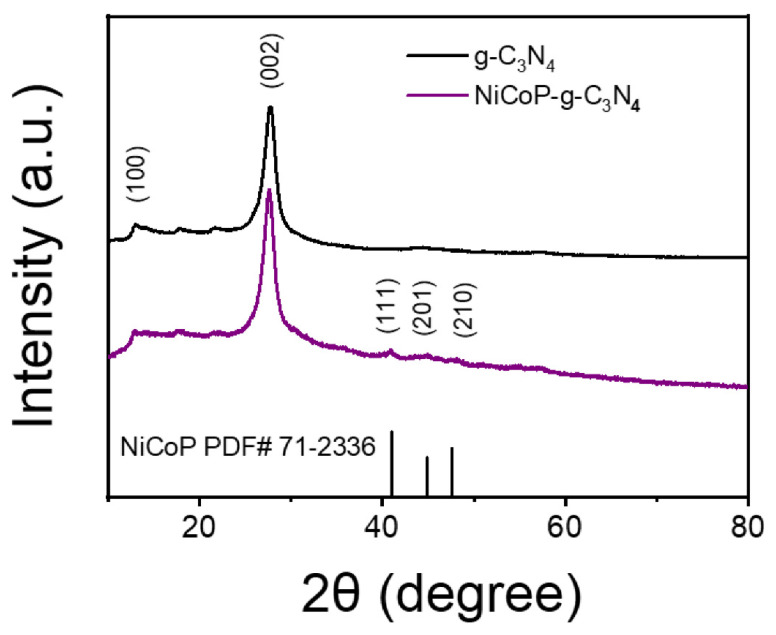
XRD patterns of g-C_3_N_4_ and NiCoP/g-C_3_N_4_ nanocomposites.

XRD was used to distinguish the phases and the structures of bare g-C_3_N_4_ and NiCoP loading. As shown in Figure 2, the lattice planes of triazine units (100) (13.1°) and the lattice planes of interlayer stacking of aromatic segments (002) (27.8°) were detected, indicating the bare g-C_3_N_4_ was successfully synthesized [35,39]. For the NiCoP/g-C_3_N_4_ samples, beside the strongest diffraction peaks of g-C_3_N_4_, several weak peaks appeared at 41°, 44.95° and 47.97°, which correspond to the (111), (201) and (210) lattice planes of NiCoP, respectively [40,41] (JCPDS No. 71-2336). These results implied that the NiCoP nanoparticles had been successfully loaded on the surface of g-C_3_N_4_ nanosheets.

**Figure 3:**
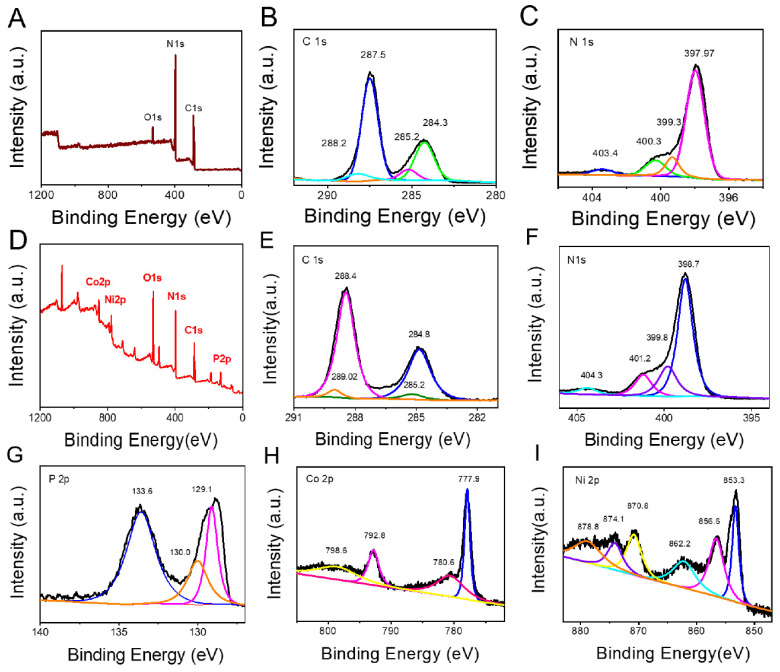
(**A**) The full XPS spectrum of g-C_3_N_4_. (**B**) The related high-resolution spectra of C 1s. (**C**) The related high-resolution spectra of N 1s. (**D**) The full XPS spectrum of NiCoP/g-C_3_N_4_. (**E**) The related high-resolution spectra of C1s. (**F**) The related high-resolution spectra of N 1s. (**G**) The related high-resolution spectra of P 2p. (**H**) The related high-resolution spectra of Co 2p. (**I**) The related high-resolution spectra of Ni 2p.

Detailed information on the g-C_3_N_4_ and NiCoP/g-C_3_N_4_ nanocomposites was obtained by XPS. The survey spectra of bare g-C_3_N_4_ and NiCoP/g-C_3_N_4_ were shown in Figure 3A,D. C, N and O elements in g-C_3_N_4_ and the C, N, P, Co and Ni elements in the NiCoP/g-C_3_N_4_ sample were detected, respectively. The detected O 1s peaks in the bare g-C_3_N_4_ were mainly due to the surface-adsorbed O_2_ and H_2_O molecules. By contrast, the O 1s peak of NiCoP/g-C_3_N_4_ increased significantly, which may have been caused by the surface oxidation of NiCoP nanoclusters [36]. Four binding energy peaks were shown in the C 1s XPS spectrum of the original g-C_3_N_4_ at 284.3, 285.2, 287.5 and 288.2 eV (Figure 3B), which originated from the graphitic, C-N/C-O bindings, cyanide/cyanoquione and heptazine typed carbons, respectively. Four peaks located at 397.97 eV, 399.3 eV, 400.3 eV and 403.4 eV were well-suited for the N 1s XPS spectra of g-C_3_N_4_ (Figure 3C), which is attributed to the heptazine N, pyrrolic N, graphitic N and oxidic N, respectively [33]. By comparing the binding energy spectrum after NiCoP deposition, we found the shift in the C 1s and N 1s spectra of NiCoP/g-C_3_N_4_ (Figure 3E,F), indicating an internal force between NiCoP and g-C_3_N_4_. For the P 2p spectrum (Figure 3G), the binding energy peak was located at 127.7 eV, which was close to the P 2p3/2, indicating the presence of the P element. Moreover, the peak at 131.9 eV could be attributed to the oxidized phosphorus species in contact with air [40,42]. For Co 2p, 777.25 eV and 792.2 eV the binding peaks were attributed to Co 2p3/2 and Co 2p1/2 of metallic Co because of the formation of Co-P [43,44] (Figure 3H). Likewise, for the Ni 2p region, the strong binding energy of 852.25 eV was close to the nickel metal (852.6 eV), which implied the presence of partially charged Ni species (Figure 3I). The XPS results further confirmed that NiCoP nanoparticles had been successfully loaded onto the g-C_3_N_4_ surface.

**Figure 4:**
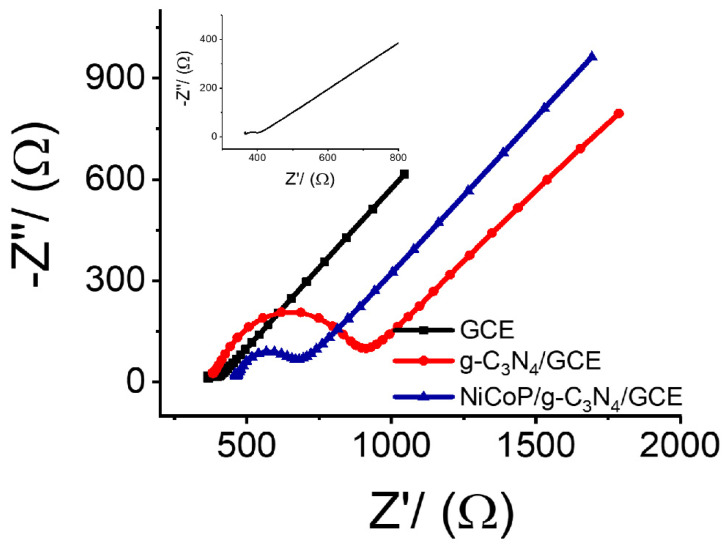
EIS spectrum of g-C_3_N_4_ and NiCoP/g-C_3_N_4_ composites. The insert is the EIS spectrum of bare electrode.

The electrochemical properties of NiCoP/g-C_3_N_4_ nanocomposites were evaluated with electrochemical impedance spectroscopy (EIS), with [Fe(CN)_6_]^3−^/[Fe(CN)_6_]^4−^ as the redox probe, and the semicircle diameter was equivalent to the electro-transfer resistance. As shown in Figure 4, compared with the bare g-C_3_N_4_, the interface electron transfer resistance (Ret) decreased significantly after the NiCoP/g-C_3_N_4_ nanocomposites were modified, indicating that the binding of NiCoP nanoparticles could change the band structure of g-C_3_N_4_ and promoted the electron transfer [45].

### 3.2. Characterization of Carboxylic NiCoP/g-C_3_N_4_ Nanocomposites

**Figure 5:**
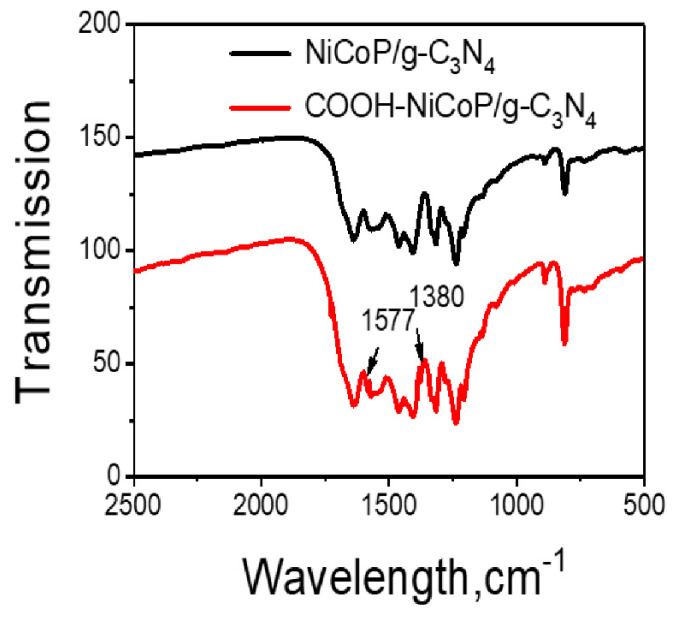
FT-IR spectroscopy of carboxylated NiCoP/g-C_3_N_4_.

The functionalization of NiCoP/g-C_3_N_4_ nanocomposites was investigated using the FT-IR spectrum. The spectral bands at different wavelengths correspond to specific vibrations of molecular functional groups. Figure 5 shows the NiCoP/g-C_3_N_4_ before (black line) and after carboxylation (red line) in the wavelength range of 500–2500 cm^−1^. The band at 806 cm^−1^ was characteristic of tri-s-triazine, and the bands in the range of 1240–1643 cm^−1^ could be attributed to the C-N stretching of g-C_3_N_4_. Furthermore, the peak at 1380 cm^−1^ and 1577 cm^−1^ corresponded to the COO bending band [46]; these results indicated that the surface of NiCoP/g-C_3_N_4_ had been successfully carboxylated.

### 3.3. Biosensor Characterization 

**Figure 6:**
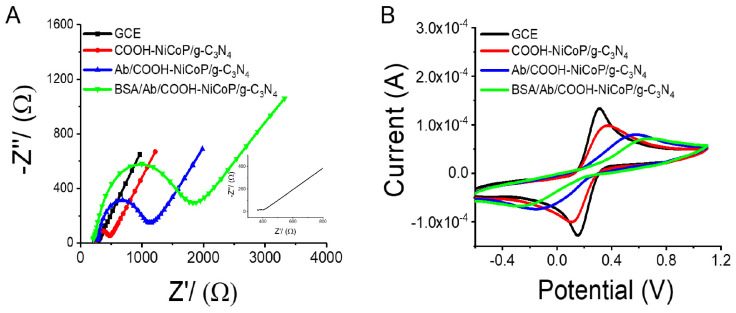
EIS spectrum (**A**) and CV (**B**) responses corresponding to electrode surface modification. Detection buffer: 1 mL 0.1M Tris-HCl containing 5 mM [Fe(CN)_6_]^3−/4−^. The insert is the EIS spectrum of bare electrode.

Cyclic voltammetry (CV) and EIS were used to verify the electrode surface modification. As shown in Figure 6 A, the bare glass carbon electrode exhibited almost a straight line, illustrating that the electro-transfer process was mainly caused by mass diffusion. The Ret increased after COOH-NiCoP/g-C_3_N_4_ nanocomposites and the antibody(Ab) were modified through π-π stacking and a zero-length amine-reactive cross-linker, EDC and NHS, respectively, which can be attributed to the COOH-NiCoP/g-C_3_N_4_ nanocomposites. The larger size of Ab blocked or hindered the diffusion of the redox probe of [Fe(CN)_6_]^3−/4−^ and eventually increased the electron transfer resistance [47]. These results were consistent with those obtained from CV measurements (Figure 6B). The above results showed that the sensing interface had been successfully fabricated.

### 3.4. Detection Performance of the Electrochemical Immunosensor

**Figure 7:**
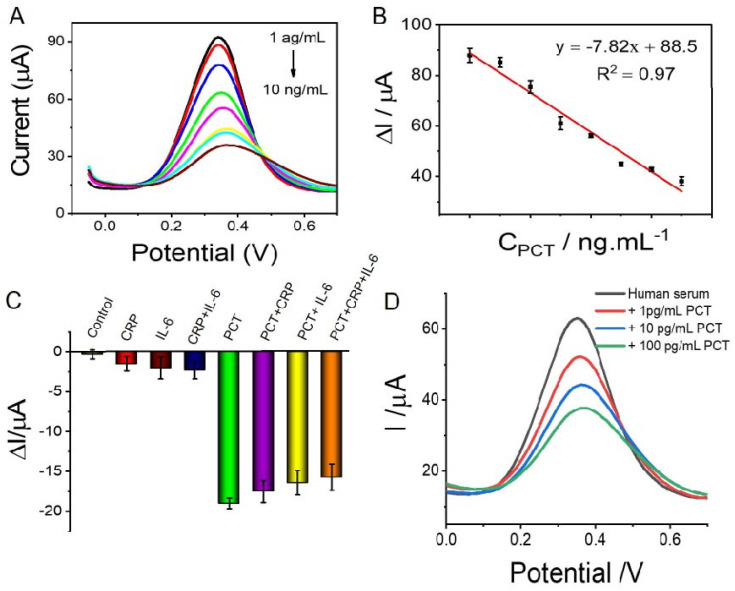
(**A**) The DPV signals of the biosensor incubated with differential concentrations of PCT. Insert: linear relationship between the increased peak current and the concentration of PCT. (**B**) Linear plot characteristics of ΔI of sensor versus the concentration of PCT in PBS buffer. (**C**) Sensor selectivity: CRP (10 pg/mL), IL-6 (10 pg/mL), PCT (1 pg/mL). (**D**) DPV curves showing continuous monitoring for PCT concentration in the human sample using NiCoP/g-C_3_N_4_/GCE.

The PCT detection performance was evaluated by exposing the sensing platform to the different concentrations of PCT under the same experimental conditions and monitored by DPV. When the PCT was captured, the electron transfer resistance on the electrode surface was enhanced, finally affecting the output of the electrochemical signal. As shown in Figure 7A, in the range of 1 ag/mL to 10 ng/mL, the electrochemical oxidation peak current dropped gradually as the PCT concentration increased. Additionally, the DPV signal and PCT concentrations showed a linear relationship; the linear equation can be described as I(μA) = 88.5–7.82 c (R^2^ = 0.97) (Figure 7B), and the limit of detection (LOD) was estimated to be 0.6 ag/mL (S/N = 3). The results illustrate that compared with other electrochemical methods, dynamic light scattering (DLS), SPR biosensors and enzyme-free immunosensor methods, as listed in Table 1, our sensor presents a competitive detection limit.

C-reaction protein (CRP) and Interleukin-6 (IL-6) were selected as interfering biomarkers to evaluate the selectivity of this immunosensor. CRP was used to indicate tissue damage inflammation [56], and IL-6 has been linked to various diseases such as inflammatory bowel disease, diabetes, osteoarthritis and asthma [57]. For specific detection, the concentration of PCT, CRP and IL-6 were 1, 10 and 10 pg/mL, respectively. As shown in Figure 7C, for each interfering substance, the DPV value was close to the control sample; however, after mixing with the PCT, the DPV response of the mixture was similar to PCT alone, indicating that our sensor can specifically recognize and capture the PCT. This means that the electrochemical sensor has high specificity and selectivity.

### 3.5. Application for the Real Sample Analysis

The practical application of NiCoP/g-C_3_N_4_ nanocomposites electrochemical PCT immunosensors was evaluated in blood PCT detection. We analyzed the PCT concentrations in human serum samples. The DPV curve in Figure 7D shows the decrease of oxidation peak current as the PCT concentrations rise from 1 pg/mL to 10 pg/mL. Through the linear relationship with the NiCoP/g-C_3_N_4_/CGE PCT sensor, the recovery of the spiked PCT (1 pg/mL, 10 pg/mL, 100 pg/mL) in 100-fold diluted human serum samples ranged from 93 to 101.2% (Table 2). These results strongly indicate that this immune sensor has great potential for analyzing PCT in real clinical samples in the future.

## 4. Conclusions

In this study, an electrochemical immunosensor using NiCoP/g-C_3_N_4_ composite as a sensor platform was constructed and utilized for PCT detection. This sensor showed excellent sensing performance, and the analytical performance of this sensing platform had the linearity range of (1 ag/mL to 10 ng/mL (R^2^ = 0.97)) and LOD of (0.6 ag/mL (S/N = 3)). At the same time, the properties of selectivity and recovery were also investigated. Those results indicated the validity of this sensing platform. In conclusion, our work presents a promising new method for PCT detection that may transform future clinical applications.

## Figures and Tables

**Table 1:** Comparison of the proposed method with previous reports on PCT detection.

Method	Linear Range	LOD	Refs.
Electrochemical	0.0001 to 100 ng/mL	0.11 pg/mL	[17]
Dynamic light scattering (DLS)	0.035 to 250 pg/mL	0.03 pg/mL	[48]
Chemiluminescence immunoassay	0.044 to 100 ng/mL	0.044 ng/mL	[49]
Electrochemical immunoassay	0.005 to 50 ng/mL	1.67 pg/mL	[50]
Immunochromatographic assay	0.49 to 13.90 ng/mL	0.1 ng/mL	[51]
Electrochemical immunosensor	0.05 to 90 pg/mL	0.014 pg/mL	[52]
SPR biosensors	25 to 1000 ng/mL	30 ng/mL	[53]
Photoelectrochemical immunoassay	0.1 pg/mL to 50 ng/mL	0.03 pg/mL	[54]
Enzyme-free immunosensor	0.25 to 250 ng/mL	0.022 ng/mL	[55]
Electrochemiluminescence	0.014 pg/mL to 40 ng/mL	3.4 fg/mL	[1]
Electrochemical immunosensor	1 ag/mL to 10 ng/mL	0.6 ag/mL	This work

**Table 2:** Recovery rate of PCT in human serum samples.

Samples	Added (pg/mL)	Found (pg/mL)	Recovery (%)	RSD (%) *
1	1	0.93	93	2.36
2	10	9.67	96.7	1.54
3	100	101.2	101.2	3.15

* RSD (%) was determined from triple parallel experiments.

## Data Availability

All data are included within the manuscript.
